# Genome Analysis of the *Janthinobacterium* sp. Strain SLB01 from the Diseased Sponge of the *Lubomirskia baicalensis*

**DOI:** 10.3390/cimb43030156

**Published:** 2021-12-11

**Authors:** Sergei I. Belikov, Ivan S. Petrushin, Lubov I. Chernogor

**Affiliations:** Limnological Institute, Siberian Branch of the Russian Academy of Sciences, 664033 Irkutsk, Russia; ivan.kiel@gmail.com (I.S.P.); lchernogor@mail.ru (L.I.C.)

**Keywords:** *Janthinobacterium* sp. SLB01, genome analysis, secretion systems, virulence factors

## Abstract

The strain *Janthinobacterium* sp. SLB01 was isolated from the diseased freshwater sponge *Lubomirskia baicalensis* (Pallas, 1776) and the draft genome was published previously. The aim of this work is to analyze the genome of the *Janthinobacterium* sp. SLB01 to search for pathogenicity factors for Baikal sponges. We performed genomic analysis to determine virulence factors, comparing the genome of the strain SLB01 with genomes of other related *J. lividum* strains from the environment. The strain *Janthinobacterium* sp. SLB01 contained genes encoding violacein, alpha-amylases, phospholipases, chitinases, collagenases, hemolysin, and a type VI secretion system. In addition, the presence of conservative clusters of genes for the biosynthesis of secondary metabolites of tropodithietic acid and marinocine was found. We present genes for antibiotic resistance, including five genes encoding various lactamases and eight genes for penicillin-binding proteins, which are conserved in all analyzed strains. Major differences were found between the *Janthinobacterium* sp. SLB01 and *J. lividum* strains in the spectra of genes for glycosyltransferases and glycoside hydrolases, serine hydrolases, and trypsin-like peptidase, as well as some TonB-dependent siderophore receptors. Thus, the study of the analysis of the genome of the strain SLB01 allows us to conclude that the strain may be one of the pathogens of freshwater sponges.

## 1. Introduction

Sponges (Porifera) are sessile metazoans, filter-feeding invertebrates that grow in many different ecological niches around the world and contain a variety of symbiotic microorganisms, including viruses, bacteria, microalgae, fungi, and protozoa [1,2]. However, in recent years, there has been a problem of disease and death of marine and freshwater sponges [3,4,5].

However, the study of diseases of sponges is difficult because they are the host organisms of a wide variety of permanent, symbiotic microbial communities that are commensals [6]. In most cases, it was unclear whether the bacteria are the primary or secondary pathogens in the disease process. Recently, it was suggested that healthy and diseased sponges were distinguished by a shift in the composition of prokaryotic communities [7,8]. A number of studies have shown the loss of the main symbionts that are characteristic of healthy sponges with the appearance of a high abundance of uncharacteristic pathogens [8,9,10]. The large number and low relative abundance of bacteria in newly colonized tissues made it difficult to find real pathogens of sponges; therefore, etiological agents were found only in a few cases [9,11,12]. Subsequently, the strain was identified by NGS sequencing and complete genome analysis as the gamma *Proteobacterium Pseudoalteromonas agarivorans* NW4327 [13,14].

The same important problem of disease and death of sponges are in the freshwater ecosystem. Lake Baikal is an ancient freshwater lake, which is home to many endemic sponges (Demosponges, Lubomirskiidae). The freshwater sponges (Lubomirskiidae) are primitive multicellular symbiotic animals with the complex consortium of many microorganisms. Over the last years, there has been mass disease and death of sponges in Lake Baikal [5,15]. Significant differences in the microbial composition were found in samples of healthy and diseased sponges *L. baicalensis* by using metagenomics analysis [5]. These differences in the structure of the community resulted in a significant decrease in the relative abundance of microorganisms, including green microalgae, the dominant eukaryotic symbionts of healthy sponges, and *Actinobacteria* [5,16]. Several families of bacteria predominated in diseased sponges, in particular *Flavobacteriaceae*, *Moraxellaceae*, and *Oxalobacteraceae* [16].

During the sequencing of metagenomes, we observed a significant increase in the number of bacteria OTU of the Burkholderiaceae family [5,16]. Strain *Janthinobacterium* sp. SLB01 of the family *Oxalobacteraceae* was isolated by culturing a bacterial suspension from a diseased sponge of the *L. baicalensis.* The draft genome of the *Janthinobacterium* sp. strain SLB01 was published [17].

It is assumed that most of the described *Janthinobacteria* species are not pathogenic, with the exception of *Janthinobacterium* *agaricidamnosum*, in which the aim of the work was the pathogen of the cultivated mushroom *Agaricus bisporus* [18] and co-incubation studies of *Janthinobacterium* sp. HH102 with the plant pathogen *Fusarium graminearum* [19]. The pathogenicity of *Janthinobacteria* in sponges has not been described; therefore, a detailed study of potential virulence factors and possible causes of pathogenicity were necessary. The cause of diseases of the Baikal sponges remains unclear. 

The aim of the work was to analyze the genome of strain *Janthinobacterium* sp. SLB01 to search for factors of the pathogenicity for sponges, which will allow the understanding of specific directions for further experimental work to determine the cause of death of the sponges.

## 2. Materials and Methods

### 2.1. Bacterial Strain and Growth Media

In this study, strain *Janthinobacterium* sp. SLB01 was isolated from samples of the diseased sponge *L. baicalensis* (collected by divers in Lake Baikal, Central Siberia, Russia). Five collected sponge samples were used to test the isolated bacterial strains in triplicate in a series of successive passages. The cell suspension was isolated from infected dirty green sponges, which were homogenized and passed through an MF-Millipore membrane filter, pore size 0.45 μm (Merck, Eysins, Switzerland), and by 10 μL added to culture dishes (TPP Techno Plastic Products AG, Trasadingen, Switzerland) with the media. The bacterial biomass was cultured on nutrient media with R2A (0.05% yeast extract, 0.05% tryptone, 0.05% casamino acids, 0.05% dextrose, 0.05% soluble starch, 0.03% sodium pyruvate, 1.7 mM K2HPO4, 0.2 mM MgSO4, final pH 7.2 adjusted with crystalline K2HPO4 or KH2PO4) agar plates (Merck KGaA, Darmstadt, Germany) at 15 °C, at pH 7.2, with the removal of fast-growing colonies. Then, in a series of successive passages, individual colonies of microorganisms were obtained. Cell morphology was determined by light microscopy Axio Imager Z2 microscope (Zeiss, Oberkochen, Germany) equipped with fluorescence optics (self-regulating, green HBO 100 filter, 490 nm excitation, 520 nm emission). The isolates of bacteria were stained with a NucBlue Live ReadyProbes reagent (Thermo Fisher Scientific Inc., Waltham, MA, USA). We observed every day for 14 days during the growth of the bacteria strain. All the images were taken with a Canon EOS 200D digital camera. One of the best strains was used for sequencing on Illumina MiSeq.

### 2.2. Genome Assembly, Annotation and Phylogenetic Relationship

We performed the genome assembly with SPAdes version 3.11.0 [20] with paired reads quality correction using FastP software [21]. To make two scaffolds from the draft assembly Ragout version 2.2 software was used [22], (https://github.com/fenderglass/Ragout accessed on 10 September 2019). Final genome was automatically annotated by NCBI during the release in GenBank.

The ribosomal gene sequences of *Janthinobacterium* sp. SLB01 was assembled from the raw reads by MATAM software with default settings [23]. A phylogenetic tree for a set of closer strains was constructed using MEGA X with the maximum likelihood method and the Tamura-Nei model [24]. A phylogenetic tree for selected strains was constructed using PhyloPhlAn based on concatenated alignments of up to 400 conserved proteins using “supermatrix_aa” and low diversity mode with the “phylophlan” database [25].

The main virulence-related genes were identified using RAST SEED pipeline [26] in categories “membrane transport” and “protein degradation”. Most of the genes related to virulence were found using NCBI annotation of the genome and by manual BLASTP search throughout the genome. Part of the virulence factors in the genome of *Janthinobacterium* sp. SLB01 were discovered with the BLASTP search against 2 databases MvirDB [27] and VFDB [28] with e-value 10^−6^ and other settings set to default. The search of homologous loci was performed with BLASTP against proteomes of the related genomes (Table 1) with e-value 10^−6^ and other settings set to default.

The genes encoding proteins of type VI secretions system (T6SS) were identified by SecReT6 (http://db-mml.sjtu.edu.cn/SecReT6/, mode T6SS-HMMER accessed on 5 August 2020) web service with other settings set to default [29]. Genes related to other secretion systems were found by BLASTP search according to previously published data, then manually checked. Protein sequences used for the search were taken from respective studies. To compare gene clusters of *Janthinobacterium* sp. SLB01 with related species (Table 1) we used BLASTP with e-value set to 1e^−6^ and other settings set to default. The presence of signal peptide in putative secreted proteins was predicted by SignalP v. 5.0 web service (http://www.cbs.dtu.dk/services/SignalP [30] accessed on 15 September 2021 for “Gram-negative” organism group).

Putative secreted protein for T6SS was predicted using Bastion6 web service [31].

## 3. Results and Discussion

### 3.1. Cultivation and General Description of the Genome of the Janthinobacterium sp. Strain SLB01

**Figure 1:**
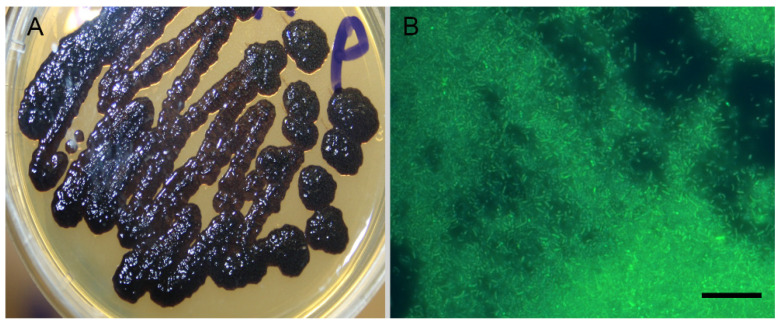
Photo image of the strain *Janthinobacterium* sp. SLB01. The bacteria with violacein pigment are visually observed. Photographs taken with Canon EOS 200D digital camera (**A**). Fluorescence microscopy of the strain *Janthinobacterium* sp. SLB01 (**B**). The sample of the bacteria with the NucBlue Live Ready Probes reagent for fluorescence microscopy. Scale bar: 10 µm.

The bacteria isolated from the diseased sponge *L. baicalensis*, which we further named *Janthinobacterium* sp. strain SLB01, was rod-shaped, Gram-negative, motile, aerobic bacteria; a purple pigment violacein appeared on the second day (Figure 1A). Colonies of the *Janthinobacterium* sp. SLB01 are single rods, 0.2–0.3 μm in diameter and 1.0–2.0 μm long, with twitching motility and flagella (Figure 1B).

The bacteria were well cultivated at 22 °C on the R2A medium. They formed many smooth translucent colonies with round edges, which were stained violet-pigmented dark purple on days 2–3 of cultivation (Figure 1A).

Genome assembly and annotation with NCBI Prokaryotic Genome Annotation Pipeline (PGAP) for *Janthinobacterium* sp. SLB01 was conducted as described previously [17]. The whole-genome sequence is available in NCBI GenBank with acc. number NZ_VZAB01000001. Genome completeness analysis with benchmarking universal single-copy orthologs (BUSCO) [32] showed that *Janthinobacterium* sp. SLB01 has 99.1% complete, no fragmented, and 0.9% missing BUSCOs. 

### 3.2. Molecular Phylogenetic Analyses of Janthinobacterium sp. Strain SLB01

**Figure 2:**
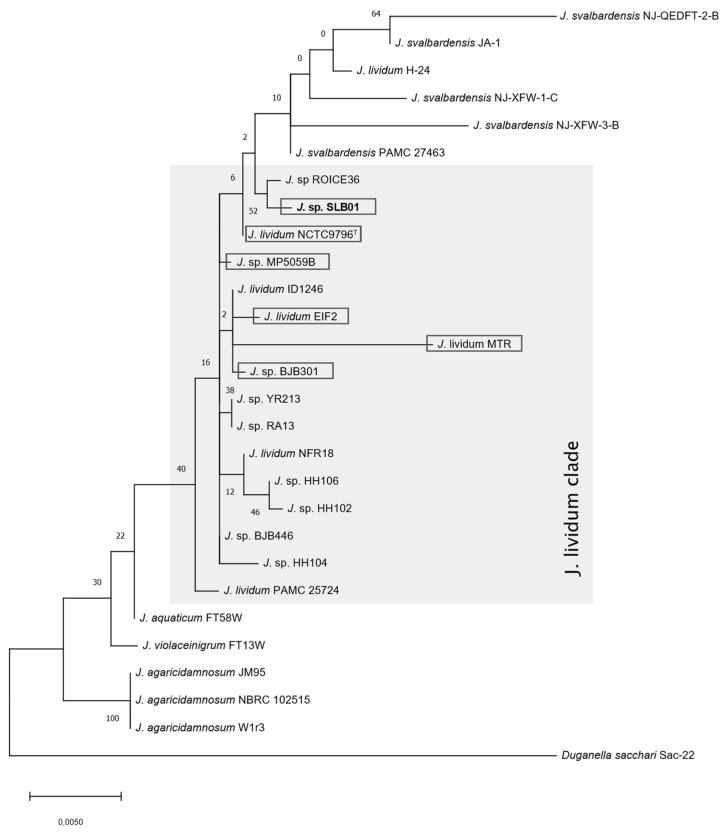
Phylogenetic tree of 16S rRNA partial sequences of *Janthinobacterium* sp. SLB01 with valid species. The tree was constructed using the maximum likelihood method and the Tamura-Nei model using MEGA X.

Phylogenetic reconstruction using 16S rRNA sequences derived from the genome sequence of *Janthinobacterium* sp. strain SLB01 and valid species within the genus *Janthinobacterium* confirmed that our strain belongs to the species *Janthinobacterium lividum*, which was closely related to *J. lividum* NCTC 9796^T^ isolated in Michigan soil [33]. The strain SLB01 was located in the *J. lividum* clade and the sequences of 16S rRNA were 99% identical (Figure 2).

According to average nucleotide identity analysis and distance matrix *Janthinobacterium* sp. SLB01 belongs to the *J.* *lividum* clade as well (Figure S1 and Table S12, closer species highlighted with gray, see Supplementary Materials). The distance between *Janthinobacterium* sp. SLB01 and the type strain *J. lividum* NCTC9796^T^ is 0.0039, although for closer species it is even smaller, from 0.0032 to 0.0034.

**Figure 3:**
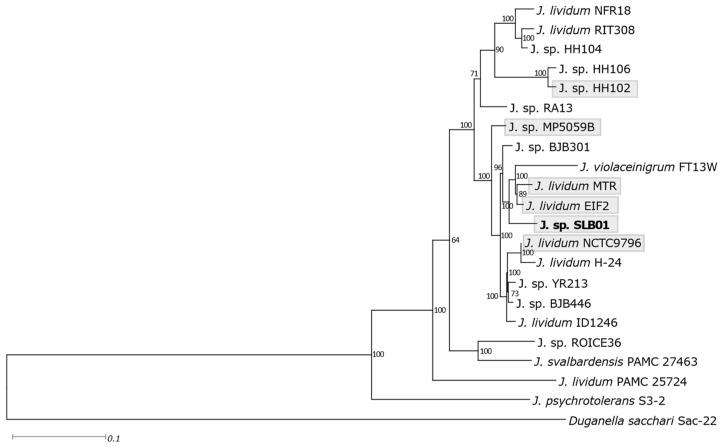
Phylogenetic tree of the strain SLB01 with closely-related species of *Janthinobacterium*. The multilocus tree was built based on a maximum-likelihood method of approximately 400 universal marker genes by PhyloPhlAn with the maximum-likelihood method.

The differences in the 16S rRNA gene sequences within *J. lividum* are insignificant, and many of them cannot be distinguished, therefore, multilocus sequence analysis was performed in this report (Figure 3). 

Analysis showed that strain *Janthinobacterium* sp. SLB01 is closest to the *J. lividum* MTR and Eif2 strains isolated respectively from Cajón del Maipo, Chile [34], and from the environmental oligotrophic water pond in Germany [35]. Both of these strains were closest (Table S12) to the strain *Janthinobacterium* sp. SLB01, which was isolated from diseased sponges, but the pathogenicity of the strains *Janthinobacterium* MTR and EIF2 in animals was not described. This fact indicates the impossibility of predicting the pathogenicity of strains on the basis of their phylogenetic proximity.

### 3.3. Secretion Systems

It is known that pathogenic organisms differ from non-pathogenic ones by the presence of genes encoding various determinants of virulence, such as adhesins, toxins, other virulence factors, and plasmids and other mobile genetic elements [36]. These factors are secreted onto the surface of a bacterial cell or into the extracellular environment using various secretion systems, which are molecular nanomachines that deliver certain proteins to the extracellular environment or directly to target cells. Depending on the target, they are introduced into the target cell, released into the extracellular space, or remain attached to the outer bacterial membrane [37]. To date, nine different types of protein secretion systems have been found in Gram-negative bacteria, including six major secretion systems (T1SS-T6SS), Chaperone-usher pathways (CU) (T7SS), curli biogenesis system (T8SS), and T9SS (PorSS) [38].

We found that the genome of the *Janthinobacterium* sp. strain SLB01 encodes all three major proteins of the TAT pathway in a single cluster, listed in Table S1, with several proteins that are secreted by the Tat pathway and may be associated with virulence. It is known that the twin-arginine translocation (TAT) system, located in the cytoplasmic membranes of many bacteria, archaea, and chloroplasts, provides active transport of export-folded proteins across the inner lipid membrane. The Tat system is required for colonization, biofilm formation, and virulence in some species [39].

The strain *Janthinobacterium* sp. SLB01 encodes all the genes required for the Sec multidimensional transport system (Table S1). We have found three peptidases, LepB, LspA, and BfpA, which are homologous to peptidases in *Escherichia coli*. Signal peptidase I (LepB) is found twice and is encoded by loci F3B38_RS12095 and F3B38_RS20245. Signal peptidase II (LspA or lipoprotein signal peptidase) is encoded by the F3B38_RS23550 locus. Signal peptidases BfpA (prepilin peptidases) are encoded by four loci F3B38_RS02270, F3B38_RS05895, F3B38_RS10150, and F3B38_RS22915 and differ significantly in amino acid sequences. The SecYEG translocase proteins are shown in Table S1. These proteins are required for a two-step secretion process to move proteins across the outer membrane [40] and also YidC and YajC proteins, which function in conjunction with Sec translocase to insert proteins into the membrane [41]. The genes and encoded proteins of the TAT and SEC secretion systems are conserved in all analyzed *Janthinobacteria* strains and the homology of proteins with the SLB01 strain exceeds 96%.

The strain *Janthinobacterium sp.* SLB01 encodes all the necessary genes for three copies of T1SS, including genes for the outer membrane protein TolC, the HlyD superfamily type I secretion periplasmic adapter subunit and type I secretion system permease/ATPase (Table S1). In addition, other genes, TolC and the HlyD superfamily, were found, which are not part of the secretion system complexes and, probably, perform other functions. 

The genes of the secretion system are conservative in all analyzed strains, but the composition of secreted proteins differs significantly in different strains. Thus, the annotated genome of the type strain *Janthinobacterium lividum* NCTC9796^T^ contains the genes for hemolysin (yqfA), three genes for hemolysin-coregulated protein (hcp, hcp1_1, hcp1_2) and one for hemolysin secretion protein D (hlyD). At the same time, the genome annotation for SLB01 contains one hemolysin III family protein, two alpha-xenorhabdolysin family binary toxin subunit A, and three ShlB/FhaC/HecB family hemolysin secretion/activation proteins. The genes of the RTX pore-forming toxin α-hemolysin and other members of the RTX toxin superfamily were not found. Probably alpha-xenorabdolysin can be secreted by the T1SS system [36]. This system allows the secretion of a wide variety of proteins, such as adhesins, proteases, and toxins from the cytoplasm into the extracellular environment [42,43]. Proteins of other strains are shown in Table S1.

The type II secretion pathway (T2SS) is the main terminal branch of the general secretion pathway (Sec secretion system), which is responsible for the extracellular secretion of toxins and hydrolytic enzymes, many of which contribute to the pathogenesis of plants and animals. The type II secretion system proteins are conserved in all analyzed strains. The proteins of pili biosynthesis are also conserved, with the exception of strain NCTC9796^T^, in which many proteins of pili biosynthesis have insignificant homology with the proteins of the strain SLB01 (Tables S1 and S6). This system was found in a wide variety of pathogenic and non-pathogenic Gram-negative bacteria. T2SS uses proteins secreted into the periplasm via the Sec or Tat system as substrates, and then transports these folded proteins through the OM and releases them into the EM [44]. This pathway has many similarities with the type IV pilus biogenesis system, including the ability to assemble a pilus-like structure. In fact, the two systems can be variations on the same secretion system [45,46]. Proteins of the T2SS secretion system are mainly conservative, the greatest number of differences was found in the HH02 strain. In addition, two T2SS proteins of the SLB01 strain (F3B38_RS18390 and F3B38_RS18395) and a prepilin-type N-terminal cleavage/methylation domain-containing protein (F3B38_RS18400) significantly differ from the corresponding proteins in other strains. Analysis of the primary amino acid sequence of proteins secreted by this pathway shows no obvious homology. 

The type III secretion system (T3SS) forms an extracellular needle to transfer substrates from a bacterial cell to a host cell or exports distal flagellar components, which constructs an extracellular flagellar filament. We did not find injectisome protein genes in the strain SLB01, but we found genes of flagella biosynthesis protein subunits. The strain *Janthinobacterium* sp. SLB01 has 42 genes encoding the proteins of the flagellar biosynthesis system (Table S1). A flagellum is a rotating, coiled filament anchored inside bacterial membranes, which allows bacteria to move through liquid media to find the optimal concentration of nutrients, oxygen, or temperature optimum [47,48]. The proteins of the T3SS secretion system are generally conserved in all strains analyzed, with the exception of one of the two flagellar filament capping proteins FliD (Table S1).

The genome of the strain *Janthinobacterium* sp. SLB01 does not contain genes encoding the type IV secretion system (T4SS), which may indicate the limited ability of the strain to transport DNA, transposons, and transposable elements. The T4SS secretion systems were found in both Gram-positive and Gram-negative bacteria and they mediate the secretion of proteins, protein toxins, and DNA nucleoprotein complexes both through the inner and outer membranes. Because T4SS is capable of transferring both DNA and proteins, it can perform many functions, including conjugative transfer of DNA (plasmids or transposons) between bacterial cells, uptake and release of DNA from the extracellular environment, and translocation of effector proteins or DNA/protein complexes directly into recipient cells [40]. The *Janthinobacterium* strains EIF2, MTR, and MP5059B also do not have T4SS proteins, but strain HH102 contains seven of them: virB1, virB4, virB5, virB8, virB9, virB10, and VirB11 of the type IV secretion system. The *Janthinobacterium lividum* NCTC9796^T^ contains VirD4, two virB4, virB9, virB10, and three virB11 proteins. The analysis of the functionality and representation of proteins in the T4SS pathway is not considered in this work.

The genome of the strain *Janthinobacterium* sp. SLB01 contains genes of the type V secretion system (T5SS), which indicates the presence and secretion of auto-transporter proteins. The strain SLB01 genome contains proteins of the Sec-translocon SecAEGY and proteins of the oligomeric complex BamABCDE and TamAB, genes of the membrane protein insertase YidC, and of the LolABCDE operon for secretion of outer membrane lipoproteins (Table S1). The presence of multiple genes of the T5SS indicates the possible presence of autotransporters in the strain *Janthinobacterium* sp. SLB01. Potential autotransporters are probably the secretion/activation proteins of hemolysin of the ShlB/FhaC/HecB family, two autotransporters of the outer membrane beta-stem protein, an adhesin-like protein containing the DUF2807 domain, and a protein containing the N-terminal filamentous hemagglutinin domain, a putative Ig domain-containing protein, an IPT/TIG domain-containing protein, and proteins of the patatin-like phospholipase family (Table S1). The putative Ig domain-containing proteins which mediate attachment to and invasion into their host were found in strains *Janthinobacterium* sp. SLB01 and *Janthinobacterium lividum* EIF2 only. The major adhesin filamentous hemagglutinin FHA was found in strains MP5059B. In the strain *Janthinobacterium* sp. HH102 were found one filamentous hemagglutinin and filamentous hemagglutinin transporter protein FhaC precursor and two filamentous hemagglutinin N-terminal domain-containing proteins were found in SLB01. IPT/TIG domain-containing proteins are the family that have an immunoglobulin-like fold and were found in strains SLB01, NCTC9796^T^, EIF2, MP5059B, and HH102. It should be noted that this list of autotransporters is hypothetical, and experimental confirmation is needed to identify proteins. 

**Figure 4:**
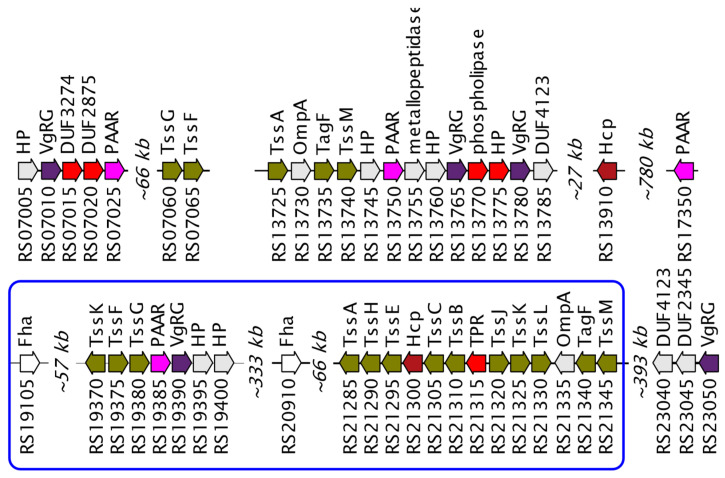
Genetic map of type VI secretion system in *Janthinobacterium* sp. SLB01 genome. The key components of T6SS are marked with navy (VgrG), brown (Hcp), and purple (PAAR) colors. The rest of T6SS components are marked with olive color. Genes encoding putative effector proteins are highlighted with bright red. Genes with unknown function and hypothetical proteins are marked with grey. The loci prefix F3B38 is omitted for short. Loci from RS07005 to RS07065 belong to the first contig, from RS13725 to RS23055 to the second contig of the whole genome.

The type VI secretion system (T6SS) is one of the most complicated systems for effectively killing competitors and is a nanomachine for introducing toxic effector proteins into prokaryotic and eukaryotic cells [49,50,51]. One large cluster of the complete system was found in the *Janthinobacterium* sp. SLB01 strain, containing all the genes encoding the type VI secretion system proteins. In addition, an incomplete operon, several orphan genes, and two FHA genes encoding a protein important for the T6SS activation mechanism [52] were found (Figure 4). 

It is known that various combinations of VgrG, PAAR and Hcp proteins can assemble functional T6SS, providing delivery of alternative sets of effectors [53]. Type VI secretion (T6SS) is a multi-protein complex, a two-membrane nanomachine with a phage tail structure [54]. A typical T6SS complex includes ~13–15 proteins that can transport substrate proteins into eukaryotic or bacterial cells and are virulence factors for many important pathogenic bacteria [55,56,57]. In addition, T6SS influences many processes, including biofilm formation [58], conjugation [59], regulation of quorum sensitivity [60], and stimulation or limitation of virulence [49,61]. 

Although the T6SS itself can be almost automatically discovered in the genome using in silico methods (Li et al., 2015), we performed the search of putative effector proteins that T6SS conduct through the cell wall. We proposed that such proteins are encoded by genes co-located with T6SS components and found five of them. These annotations and loci numbers are DUF3274 (F3B38_RS07015), DUF2875 (F3B38_RS07020), phospholipase (F3B38_RS13770), hypothetical protein (F3B38_RS13775) and tetratricopeptide repeat protein (TPR, F3B38_RS21315). T6SS proteins are present in all analyzed strains, but their composition differs significantly between strains. Thus, the T6SS proteins of the NCTC9796^T^ strain differ insignificantly, and the greatest differences were found in the HH102 strain. Differences in the structure of VgrG and PAAR domain-containing proteins may indicate differences in the virulent potentials of the T6SS system in different strains. The structure of the effector proteins of the T6SS system is not known in *Janthinobacterium*, therefore it is not possible to compare the virulent properties caused by this system.

The operon encoding the chaperone-usher pathway (CU) in the strain *Janthinobacterium* sp. SLB01 has both genes encoding the usher and chaperone proteins (Table S1), and code two spore coat U domain-containing proteins similarly to the *Myxococcus xanthus* operon [62], instead of pilus subunits. It is known that many bacteria use the chaperone-usher (CU) secretion pathway containing usher subunits, chaperone, and pili subunits to assemble fimbrial organelles on their surface, which play an important role in bacterial adhesion and biofilm formation [63]. Spore formation in *Janthinobacterium* has not been described, so the function of the chaperone-usher pathway of *Janthinobacterium* sp. SLB01 was not known.

In addition, the strain *Janthinobacterium* sp. SLB01 contains the Tol-Pal secretion system as one operon in the complementary strand (Table S1). It is known that the Tol-Pal system is necessary for the pathogenesis and virulence of many Gram-negative bacteria. The system Tol-Pal is associated with three layers of the cell wall and plays an important role in the narrowing of the outer membrane during cell division due to the active accumulation of the peptidoglycan-binding lipoprotein Pal of the outer membrane at division sites by the Tol system [64,65].

Besides, it is known the TonB-dependent porins or TonB receptors receive extracellular signals in very low concentrations and transmit them to the cytoplasm [66].

These receptors can transport iron siderophore complexes or other large substrates into the cells of Gram-negative bacteria. The *Janthinobacterium* sp. SLB01 genome encodes 50 TonB-dependent receptors, 27 TonB-dependent siderophore receptors, five energy transducer TonB, five ExbD proteins, and the ExbD/TolR family protein, one TonB-system energizer ExbB and two MotA/TolQ/ExbB proton channel family proteins. We found six biopolymer transporter ExbD and six iron ABC transporter permease (Table S1). The process of transport of substrates requires the interaction of many proteins, including TonB-dependent receptors, the complex of three proteins TonB-ExbB-ExbD, a plug domain containing proteins and a number of other proteins, such as anti-sigma factor [67,68]. Only one-third of the receptors were siderophore receptors in the strain SLB01, and the function of the rest is unknown and requires special analysis. 

### 3.4. Biofilm and Motility

A biofilm is an aggregate of microbial cells embedded in an extracellular polymer matrix and living at the interface between the liquid phase and the surface. Most of the biofilm biomass consists of hydrated extracellular polymeric substances rather than microbial cells [69]. Biofilms are bacterial analogs of a multicellular organism and make bacteria resistant to environmental conditions and antibacterial drugs [70]. Extracellular polymeric substances are organic polymers consisting mainly of lipopolysaccharides and exopolysaccharides, as well as proteins, extracellular DNA (eDNA), and lipids [71,72]. In Gram-negative bacteria, the exact relationship between lipopolysaccharides (LPS) and the outer membrane is not known, and the processes involved in the biosynthesis and export of LPS and EPS are difficult to distinguish [73]. The *Janthinobacterium* sp. SLB01 genome has a lipopolysaccharide transport pathway conserved among Gram-negative bacteria [73,74] and contains eight genes scattered across the genome (Table S2)

It is known that the number of possible EPS structures is practically unlimited [75]. The methods of assembly and secretion of bacterial exopolysaccharides from the cell are much more complicated and they can be classified into three different mechanisms: Wzx/Wzy-, ATP-binding cassette (ABC) transport-dependent pathways, and synthase-dependent pathway [76,77]. The genome of the strain SLB01 contains genes encoding proteins for the synthesis and secretion of polysaccharides using the Wzx/Wzy-dependent pathway. Some proteins of this secretion pathway are shown in Table S2. The proteins of lipopolysaccharide transport do not differ significantly from the proteins of the SLB01 strain, but the EPS transport proteins essentially differ in two strains, in the type strain NCTC9796^T^ and HH102 strain, and their homology does not exceed 80% relative to the strain SLB01 (Table S2).

In addition, it was found that the SLB01 strain contains most of the proteins of the pathway for the synthesis of branched polysaccharides, which were previously found in *Burkholderia cepacia* [78]. However, homologs of four BceGHJK glycosyltransferases were not found; instead, the 18 other nonhomologous glycosyltransferases are encoded in the genome of strain SLB01. (Table S2). It is possible that some of these glycosyltransferases can participate in the assembly of cepacian-like polysaccharide, but the structure of the repeating oligosaccharide blocks may differ significantly from those found in *Burkholderia cepacia.* It is possible that some of these glycosyltransferases can participate in the assembly of chain-like polysaccharides, but the structure of repeating blocks of oligosaccharides may differ significantly from those of *B. cepacia*. The operon of biosynthesis of the cepacian-like exopolysaccharide in the strain SLB01 contains 32 genes encoding proteins, but of them, only 14–15 proteins are homologous to the proteins of other analyzed strains. This fact suggests that *Janthinobacterium* sp. SLB01 can synthesize a unique polysaccharide that differs from the polysaccharides in *B. cepacia* and other analyzed strains.

The cepacian was composed of 5 acetylated monosaccharides of D-glucose, D-mannose, D-rhamnose, D-glucuronic acid, and D-galactose and repetitive heptasaccharide units are exported using the Wzy-dependent pathway [79]. Determination of the composition of polysaccharides in the *Janthinobacterium* sp. requires special detailed experimental verification. 

The strain *Janthinobacterium* sp. SLB01 is able to form a biofilm not only on hard surfaces but also to form suspensions of cell aggregates (flocs) floating in the water. The strain SLB01 contains a large cluster of genes for the exopolysaccharides biosynthesis, as well as a cluster of genes of PEP-CTERM proteins encoded in the *Zoogloea resiniphila* genome, which contain the two-component PrsK-PrsR system and RpoN-CTERM proteins/XrtA (Table S3). Such flocs, which represent a special type of bacterial aggregation, have been described mainly for activated sludge bacteria [80]. The formation of such bulky particles by the strain SLB01 can probably facilitate the colonization of the sponge’s aquifer system, blocking the narrow internal channels with the formation of a kind of “clots”. Such clots can mechanically restrict microcirculation, affecting the delivery of nutrients and reducing the rate of removal of waste products from the sponge. Such blockage can also facilitate the attachment of other microorganisms, for example, *Pseudomonas aeruginosa* and *Bacillus cereus* [81] on the inner surface of the sponges, increasing their toxic effect [82]. Inside such “clots”, consisting of the *Janthinobacteria*, the concentration of autoinducers of quorum sensing can locally increase, which can lead to the activation of virulent properties and, as a consequence, to the destruction of sponge tissues. 

The flocs formation in *Janthinobacrerium* has not been previously observed, although genes encoding the synthesis of exopolysaccharides and PEP-CTERM proteins required for floc formation in *Z. resinaiphila* [80,83] have been found in all analyzed *Janthinobacterium* strains. It cannot be ruled out that the flocculation process is more complex and that the specified PEP-CTERM proteins and exopolysaccharides are necessary, but not sufficient, for effective floc formation.

The *Janthinobacterium* sp. strain SLB01 has a large set of genes for the biosynthesis of polar and lateral flagella (Table S4). Flagella-based motility is the main route of movement of bacteria in the environment [84]. Flagella are unbranched, long structures made up of three distinct parts, including an elongated filament composed primarily of the flagellin protein, the hook, and the basal body, embedded in the cell membrane and serving as a motor. Many bacterial species express single or multiple polar flagella, however, a limited number of bacteria possess dual flagellar systems, for example, *Vibrio parahaemolyticus* and *Aeromonas* sp., and are able to express polar flagella for swimming and lateral flagella for swarming, which is induced upon attachment to the surfaces of host cells [85]. Bacteria can perceive a wide range of environmental signals, such as salt, nutrient or toxin concentrations, oxygen levels, pH, and light. Mobility and chemotaxis are required by many pathogenic species to colonize and invade the cell of the host.

All bacteria in which motility has been studied have chemotaxis and the strain SLB01 has a chemosensory system similar to that described for *E coli* receptors, including CheABCDRVY proteins, seven chemotaxis protein CheW, and six methyl-accepting chemotaxis proteins involved in the transmission of sensory signals from the chemoreceptors to the flagellar motors (Table S5). Chemotaxis plays an important role in bacterial ecology including flagellar biosynthesis, exopolysaccharide, and toxin production, and is required for host colonization [86,87,88]. 

The strain *Janthinobacterium* sp. SLB01 also has two sets of pili, type IVa and type IVb, which differ in the structure of the main protein, pilin (Table S6): one locus-encoding pilin, which has a signal sequence characteristic of type IVa pili [89], and four loci encoding an Flp family type IVb. Type IVb pilins were found almost exclusively on enteric pathogens, although *Janthinobacterium* has never been observed to be an animal pathogen. The locus encoding type IV pilus twitching motility protein PilT and corresponding PilT/PilU family type 4a pilus ATPase locus was also found. Twitching motility is a flagellar-independent form of bacterial movement across various surfaces by lengthening type 4 pili, attachment, and subsequent contraction [90]. 

### 3.5. Secondary Metabolite

Secondary metabolites are one of the main virulence factors and the main secondary metabolite of the *Janthinobacterium* is violacein, a bisindole, which is also produced by various bacterial genera, including *Duganella*, *Collimonas*, *Pseudoalteromonas,* and others [91]. The most famous and studied is the *Chromobacterium violaceum* [92]. Violacein synthesis is regulated by quorum-sensing mechanisms and is associated with biofilm formation [93]. 

The strain *Janthinobacterium* sp. SLB01 contains a violacein biosynthetic gene cluster with five contiguous genes vioA, vioB, vioC, vioD, and vioE, which are transcribed in the same direction (Table S7). It is known that violacein has a variety of biological activities including antiplasmodic, trypanocidal, and anti-cancer [94], including antibacterial properties against Gram-positive bacterial strains, for example against multidrug-resistant *Staphylococcus aureus* [95]. The effect of violacein on sponge cells has not been studied. However, it can be assumed that the ability of violacein to inhibit the growth of Gram-positive bacteria can lead to an imbalance in the natural composition of sponge symbionts and affect their health and facilitate the colonization of sponges by pathogenic microflora. The strain *Janthinobacterium* sp. SLB01 was isolated from diseased sponges and a sharp decrease in the number of symbiotic Gram-positive bacteria, in particular the *Actinobacteria*, was noted [5,16]. 

The antibiotic tropodithietic acid (TDA) is produced by bacteria of the marine clade *Roseobacter*, such as *Phaeobacter gallaeciensis*, *P. inhibens*, *Silicibacter* sp., and others. Orthologous genes for tropodithietic acid biosynthesis were identified in the genome of the strain SLB01 (Table S7). The TDA exhibits potent antibiotic activity against a wide range of bacteria, including alpha- and gamma-proteobacteria, *Flavobacteria*, and *Actinobacteria* [96]. It can be assumed that tropodithietic acid-like antibiotics can enhance the virulent properties of violacein during their joint production in the strain SLB01.

Another potential antibiotic in the strain *Janthinobacterium* sp. SLB01 may be marinocine, a broad-spectrum antibacterial protein. The genome of the strain SLB01 contains two operons located in the main and complementary chains and encodes the homologs of these proteins (Table S7). The marinocine synthesis operon in the lysogenic bacterium *Marinomonas mediterranea* contains two genes encoding lysine oxidase LodA and flavoprotein dehydrogenase LodB [97]. Marinocine causes the death of bacterial cells during biofilm decay, which may be associated with the formation of hydrogen peroxide [98,99]. However, the homology of the proteins found in the strain SLB01 does not exceed 30% relative to the described LodA and LodB; therefore, their biological properties may differ greatly from those described above. Such differences in activity may be interesting for the search for potential new antibiotics. The genes and proteins of TDA and marinocine biosynthesis are conserved in most of the analyzed strains, except for the *Janthinobacterium* strain HH102 (Table S7). It can be assumed that TDA and marinocine are not synthesized by this strain.

### 3.6. Quorum Sensing

Quorum sensing (QS) is a process of intercellular communication that determines the density of bacterial cells, which allows synchronization of population behavior. This process involves the production, secretion, and detection of autoinducers (AIs)—Small signaling molecules of various structures. A gene cluster encoding quorum-sensing autoinducer CAI-1 synthase (cqsA), HAMP domain-containing histidine kinase, and response regulator was found in the genome of the strain *Janthinobacterium* sp. SLB01 and these proteins had a fairly large homology with the corresponding proteins of the *Janthinobacterium* sp. HH01 [100] (Table 2).

It has recently been shown [19,100] that some *Janthinobacterium* strains encoded a type of autoinduction that was previously found in the human pathogens *Vibrio cholerae* and *Legionella pneumophila*. The *L. pneumophila* gene cluster synthesizes and detects the autoinducer 3-hydroxypentadecan-4-one, and *V. cholerae*—The autoinducer 3-hydroxytridecan-4-one [101,102]. The homology of proteins of this operon in all analyzed bacterial strains was high compared to the SLB01 strain, with the exception of the HH102 strain, but was significantly lower compared to *Legionella pneumophila* (Table 2). In this regard, it can be assumed that the structure of the autoinducer in all *Janthinobacterium* sp. may be the same, but, probably, may differ significantly from the inducers identified in *L. pneumophila*. Therefore, experimental identification of the structure of the autoinducer in the strain SLB01 is required. The structures of the autoinducer must be determined for the development of methods for inactivating QS communication and reducing the pathogenicity of *Janthinobacterium.*

### 3.7. Comparison of Virulence Factors

We compared our *Janthinobacterium* sp. SLB01 with closely-related *J. lividum* strains selected for comparison of genomic features in Table 1. The strains were isolated from soil and freshwater sources.

All analyzed *J. lividum* strains had a wide range of orthologous genes encoding highly homologous hydrolytic enzymes that may be associated with pathogenicity. In particular, found alpha-amylase, caspase family protein, chitinase, hemolysin III family, ShlB/FhaC/HecB family hemolysin secretion/activation, phospholipase ACD, triacylglycerol lipase, and collagenase. In general, the analyzed strains can be ranked in the order *Janthinobacterium* SLB01, MTR > MP5059B > EIF2 > NCTC9796 >> HH102 according to the degree of similarity of the analyzed genes (Table S8).

An incomplete list of proteins that differ from the proteins of the SLB01 strain includes alpha/beta hydrolase, glycoside hydrolases family 3, 32, 43, and 127, serine hydrolase, S8 family serine peptidase, SOS response-associated peptidase glycoside hydrolase family 32, 43b 127, serine hydrolase, SGNH/GDSL hydrolase family, SOS response-associated peptidase, trypsin-like peptidase, and others. Proteins of the caspase family and phosphocholine-specific phospholipase C were both conserved and unique depending on the strains. Half of the listed proteins had a signal sequence and were secreted, i.e., they could be associated with pathogenicity for strain SLB01 (Table S9).

Significant differences with respect to the strain SLB01 were found in the composition of TonB-dependent receptors and TonB-dependent siderophore receptors (Table S1), and the greatest differences were found in the structures of glycosyltransferases and other genes for polysaccharide biosynthesis, for example, in the supposed operon of synthesis of cepacian-like exopolysaccharides (Table S2).

The homology of proteins of the toxin-antitoxin systems in the analyzed genomes of *J. lividum* differed from the strain *Janthinobacterium* sp. SLB01 and was insignificant for all proteins of the type II toxin-antitoxin system RelE/ParE family toxin (Table S10). The RelE is a ribosome-dependent mRNA endoribonuclease that inhibits translation, while ParE acts by inhibiting DNA gyrase, converting supercoiled plasmid DNA into a linear form [103]. 

Conserved multidrug resistance and transporter family protein were in all analyzed strains, with the exception of multidrug efflux SMR transporter proteins, the identity of which was close in the strains *Janthinobacterium* sp. SLB01 and *J. lividum* MTR, but did not exceed 50% in other strains (Table S11). It is known that multidrug resistance small efflux protein removes various cationic antibiotics, disinfectants, or dyes from bacterial cells [104].

## 4. Conclusions

A high relative abundance of Janthinobacterium was found in diseased sponges of Lake Baikal. The strain *Janthinobacterium* sp. SLB01 was isolated and its pathogenicity for sponges was confirmed experimentally in accordance with Koch’s postulates (in press). The complete genome of this strain was analyzed in comparison with other published genomes of *Janthinobacterium* strains to identify the causes of the pathogenicity of this strain.

In this study, phylogenetic analysis showed that the *Janthinobacterium* sp. SLB01 belongs to the *J. lividum* cluster but belongs to the sister group relative to the group including the type strain *J. lividum* NCTC9796^T^. In a comparative analysis of its complete genome with the genomes of closely-related strains to search for pathogenicity factors, it was revealed that most virulence factors are conservative in all analyzed strains. *J. lividum* strains are considered non-pathogenic; therefore, information on the pathogenicity factors of strains is rare.

We found that the main features of strain SLB01 are associated with proteins of polysaccharide biosynthesis. Strain SLB01 is capable of producing floc, the production of which is probably due to the synthesis of branched polysaccharides. Operons associated with floc formation have been investigated for *Zoogloea resiphila*, for which flocculation is a hallmark. 

The floc formation ability in other *J. lividum* strains has not been described, although the corresponding operon and encoded proteins were identified in all analyzed *Janthinobacteria* strains and are homologous to *Zoogloea resiphila* proteins.

It can be assumed that the strain SLB01 colonizes the surface of the sponge and also can block the channels of the aquifer system due to the formation of flocs in combination with adhesion factors.

Violacein production, regulated by quorum sensitivity, can kill Gram-positive bacteria, which can lead to imbalances in symbiont composition and affect sponge health. Another virulence factor could be the T6SS secretion system nanomachine, which kills adjacent bacterial and eukaryotic cells, promoting the propagation of *Janthinobacteria*.

Then, according to this scenario, the weakened sponge organism could be attacked by opportunistic pathogens of the sponge or possibly by pathogens from the water column. In this case, the visible signs of sponge disease can vary significantly not only between individuals, but also between different parts of the sponge, depending on the types of secondary pathogens, their relative abundance, and growth rate. Various signs of the disease are characteristic of the Baikal sponges *L. baicalensis.*

## Figures and Tables

**Table 1:** List of the *Janthinobacterium* strains analyzed in this study.

Strain	Isolation Source	Genome Size, Mbases	GC Content	CDS Count	GenBank Accession Number
*Janthinobacterium* sp. SLB01	Diseased sponge, Lake Baikal, Russia	6.47	62.30	5498	VZAB01000000
*Janthinobacterium lividum* EIF2	Water pond, Göttingen, Germany	6.76	62.41	5888	NZ_CP049828
*Janthinobacterium* sp. MP5059B	Soil, Germany	6.46	62.70	5661	LRHX00000000
*Janthinobacterium lividum* strain MTR	Cajon del Maipo, Santiago Metropolitan Region, Chile	6.54	62.40	5780	JRRH01000000
*Janthinobacterium lividum* strain NCTC9796^T^	Soil, Michigan state, USA	6.76	62.40	5826	UGJH01000001
*Janthinobacterium* sp. HH102	Water rainwater-tap Botanic Garden, Hamburg, Germany	6.68	62.33	5939	NZ_CP062062

**Table 2:** Quorum sensing of the genes of *Janthinobacterium* sp. SLB01 in comparison with related species.

*Janthinobacterium* sp. SLB01		*J. liv.* MTR% Id	*J.* sp. MP5059B% Id	*J.* sp. HH102% Id	*J. liv.* EIF2% Id	*J. liv.* NCTC9796% Id	*J.* sp. HH01% Id	*L. pneumophila* % Id
Locus Tag	Gene							
F3B38_RS23475	cqsA	99	100	74	100	99	71	41
F3B38_RS23480	(cqsS)	99	99	67	99	100	62	35
F3B38_RS23485	***	99	99	97	99	99	70	33

Note: *L. pneumophila*: strain FDAARGOS_779; ID: the extent to which two sequences have the same residues at the same positions in alignment; (cqsS): HAMP domain-containing histidine kinase; ***: response regulator.

## Data Availability

This full-genome shotgun project has been deposited at DDBJ/ENA/GenBank and in the Sequence Read Archive (SRA) under BioProject number PRJNA565495, published with accession number VZAB00000000.

## Supplementary Materials

The following are available online at https://www.mdpi.com/article/10.3390/cimb43030156/s1.

## References

[1] Hentschel U., Usher K.M., Taylor M.W. (2006). Marine sponges as microbial fermenters. FEMS Microbiol. Ecol..

[2] Taylor M.W., Thacker R.W., Hentschel U. (2007). Evolutionary insights from sponges. Science.

[3] Stabili L., Cardone F., Alifano P., Tredici S.M., Piraino S., Corriero G., Gaino E. (2012). Epidemic mortality of the sponge *Ircinia variabilis* (Schmidt, 1862) associated to proliferation of a *Vibrio bacterium*. Microb. Ecol..

[4] Olson J.B., Thacker R.W., Gochfeld D.J. (2014). Molecular community profiling reveals impacts of time, space, and disease status on the bacterial community associated with the Caribbean sponge *Aplysina cauliformis*. FEMS Microb. Ecol..

[5] Belikov S., Belkova N., Butina T., Chernogor L., Kley A.M., van Nalian A., Rorex C., Khanaev I., Maikova O. (2019). Diversity and shifts of the bacterial community associated with Baikal sponge mass mortalities. PLoS ONE.

[6] Thomas T., Moitinho-Silva L., Lurgi M., Björk J.R., Easson C., Astudillo-García C., Olson J.B., Erwin P.M., López-Legentil S., Luter H. (2016). Diversity, structure, and convergent evolution of the global sponge microbiome. Nat. Commun..

[7] Angermeier H., Kamke J., Abdelmohsen U.R., Krohne G., Pawlik J.R., Lindquist N.L., Hentschel U. (2011). The pathology of sponge orange band disease affecting the Caribbean barrel sponge *Xestospongia muta*. FEMS Microbiol. Ecol..

[8] Gao Z.-M., Wang Y., Tian R.-M., Lee O.O., Wong Y.H., Batang Z.B., Al-Suwailem A., Lafi F.F., Bajic V.B., Qian P.-Y. (2015). Pyrosequencing revealed shifts of prokaryotic communities between healthy and disease-like tissues of the Red Sea sponge *Crella cyathophora*. Peer J..

[9] Webster N.S., Negri A.P., Webb R.I., Hill R.T. (2002). A spongin-boring alpha proteobacterium is the etiological agent of disease in the Great Barrier Reef sponge, *Rhopaloeides odorabile*. Mar. Ecol. Prog. Ser..

[10] Blanquer A., Uriz M.J., Cebrian E., Galand P.E. (2016). Snapshot of a bacterial microbiome shift during the early symptoms of a massive sponge die-off in the Western Mediterranean. Front. Microbiol..

[11] Rützler K. (1988). Mangrove sponge disease induced by cyanobacterial symbionts: Failure of a primitive immune system?. Dis. Aquat. Org..

[12] Vacelet J., Vacelet E., Gaino E., Gallissian M.F., van Soest R.W.M., van Kempen T.M.G., Braekman J.C. (1994). Bacterial attack of spongin skeleton during the 1986–1990 Mediterranean sponge disease. Sponges in Time and Space.

[13] Choudhury J.D., Pramanik A., Webster N.S., Llewellyn L.E., Gachhui R., Mukherjee J. (2014). Draft genome sequence of *Pseudoalteromonas* sp. strain NW 4327 (MTCC 11073, DSM 25418), a pathogen of the great barrier reef sponge *Rhopaloeides odorabile*. Genome Announc..

[14] Choudhury J.D., Pramanik A., Webster N.S., Llewellyn L.E., Gachhui R., Mukherjee J. (2015). The pathogen of the Great Barrier Reef sponge *Rhopaloeides odorabileis* a new strain of *Pseudoalteromonas agarivorans* containing abundant and diverse virulence-related genes. Mar. Biotechnol..

[15] Khanaev I.V., Kravtsova L.S., Maikova O.O., Bukshuk N.A., Sakirko M.V., Kulakova N.V., Butina T.V., Nebesnykh I.A., Belikov S.I. (2018). Current state of the sponge fauna (Porifera: Lubomirskiidae) of Lake Baikal: Sponge disease and the problem of conservation of diversity. J. Great Lakes Res..

[16] Chernogor L., Klimenko E., Khanaev I., Belikov S. (2020). Microbiome analysis of healthy and diseased sponges *Lubomirskia baicalensis* by using cell cultures of primmorphs. Peer J..

[17] Petrushin I.S., Belikov S.I., Chernogor L.I. (2019). Draft genome sequence of *Janthinobacterium* sp. strain SLB01, isolated from the diseased sponge *Lubomirskia baicalensis*. Microbiol. Resour. Announc..

[18] Lincoln S.P., Fermor T.R., Tindall B.J. (1999). *Janthinobacterium agaricidamnosum* sp. nov., a soft rot pathogen of *Agaricus bisporus*. Int. J. Syst. Bacteriol..

[19] Haack F.S., Poehlein A., Kröger C., Voigt C.A., Piepenbring M., Bode H.B., Daniel R., Schäfer W., Streit W.R. (2016). Molecular keys to the *Janthinobacterium* and *Duganella* spp. interaction with the plant pathogen *Fusarium graminearum*. Front. Microbiol..

[20] Chen S., Zhou Y., Chen Y., Gu J. (2018). Fastp: An ultra-fast all-in-one FASTQ preprocessor. Bioinformatics.

[21] Nurk S., Bankevich A., Antipov D., Gurevich A.A., Korobeynikov A., Lapidus A., Prjibelski A.D., Pyshkin A., Sirotkin A., Sirotkin Y. (2013). Assembling single-cell genomes and mini-metagenomes from chimeric MDA products *J*. Comput. Biol..

[22] Kolmogorov M., Armstrong J., Raney B.J., Streeter I., Dunn M., Yang F., Odom D., Flicek P., Keane T.M., Thybert D. (2018). Chromosome assembly of large and complex genomes using multiple references. Genome Res..

[23] Pericard P., Dufresne Y., Couderc L., Blanquart S., Touzet H. (2017). MATAM: Reconstruction of phylogenetic marker genes from short sequencing reads in metagenomes. Bioinformatics.

[24] Kumar S., Stecher G., Li M., Knyaz C., Tamura K. (2018). MEGA X: Molecular evolutionary genetics analysis across computing platforms. Mol. Biol. Evol..

[25] Segata N., Börnigen D., Morgan X.C., Huttenhower C. (2013). PhyloPhlAn is a new method for improved phylogenetic and taxonomic placement of microbes. Nat. Commun..

[26] Overbeek R., Olson R., Gordon R., Gary D.P., Olsen J., Davis J.J., Terry D.T., Edwards R.A., Gerdes S. (2014). The SEED and the rapid annotation of microbial genomes using subsystems technology (RAST). Nucleic Acids Res..

[27] Zhou C.E., Smith J., Lam M., Zemla A., Dyer M.D., Slezak T. (2007). MvirDB—A microbial database of protein toxins, virulence factors and antibiotic resistance genes for bio-defence applications. Nucleic Acids Res..

[28] Liu B., Zheng D., Jin Q., Chen L., Yang J. (2019). VFDB A comparative pathogenomic platform with an interactive web interface. Nucleic Acids Res..

[29] Li J., Yao Y., Xu H.H., Hao L., Deng Z., Rajakumar K., Ou H.-Y. (2015). SecReT6: A web-based resource for type VI secretion systems found in bacteria. Environ. Microbiol..

[30] Almagro Armenteros J.J., Tsirigos K.D., Sønderby C.K., Petersen T.N., Winther O., Brunak S., von Heijne G., Nielsen H. (2019). SignalP 5.0 improves signal peptide predictions using deep neural networks. Nat. Biotechnol..

[31] Wang J., Yang B., André Leier A., Marquez-Lago T.T., Hayashida M., Rocker A., Zhang Y., Akutsu T., Chou K., Strugnell R.A. (2018). Bastion6: A bioinformatics approach for accurate prediction of type VI secreted effectors. Bioinformatics.

[32] Waterhouse R.M., Seppey M., Simao F.A., Manni M., Ioannidis P., Klioutchnikov G., Kriventseva E.V., Zdobnov E.M. (2018). BUSCO applications from quality assessments to gene prediction and phylogenomics. Mol. Biol. Evol..

[33] De Ley J., Segers P., Gillis M. (1978). Intra-and intergeneric similarities of *Chromobacterium* and *Janthinobacterium* ribosomal ribonucleic acid cistrons. Int. J. Syst. Bacteriol..

[34] Valdes N., Soto P., Cottet L., Alarcon P., Gonzalez A., Castillo A., Corsini G., Tello M. (2015). Draft genome sequence of *Janthinobacterium lividum* strain MTR reveals its mechanism of capnophilic behavior. Stand. Genom. Sci..

[35] Friedrich I., Hollensteiner J., Schneider D., Poehlein A., Hertel R., Daniel R. (2020). First complete genome sequences of *Janthinobacterium lividum* EIF1 and EIF2 and their comparative genome analysis. Gen. Biol. Evol..

[36] Sarowska J., Futoma-Koloch B., Jama-Kmiecik A. (2019). Virulence factors, prevalence and potential transmission of extraintestinal pathogenic *Escherichia coli* isolated from different sources: Recent reports. Gut Pathog..

[37] Desvaux M., Hebraud M., Talon R., Henderson I.R. (2009). Outer membrane translocation: Numerical protein secretion nomenclature in question in mycobacteria. Trends Microbiol..

[38] Hui X., Chen Z., Lin M., Zhang J., Hu Y., Zeng Y., Cheng X., Ou-Yang L., Sun M., Aaron P. (2020). T3SEpp: An Integrated Prediction Pipeline for Bacterial Type III Secreted Effectors. mSystems.

[39] Tomich M., Planet P.J., Figurski D.H. (2007). The tad locus: Postcards from the widespread colonization island. Nat. Rev. Microbiol..

[40] Green E.R., Mecsas J. (2016). Bacterial secretion systems: An overview. Microbiol. Spectr..

[41] Dalbey R.E., Kuhn A., Zhu L., Kiefer D. (2014). The membrane insertase YidC. Biochim. Biophys. Acta Mol. Cell Res..

[42] Smitha Rao C.V., Anne J. (2011). Bacterial type I signal peptidases as antibiotic targets. Future Microbiol..

[43] Thomas S., Holland I.B., Schmitt L. (2014). The Type 1 secretion pathway—The hemolysin system and beyond. Biochim. Biophys. Acta Mol. Cell Res..

[44] Von Tils D., Blädel I., Schmidt M.A., Heusipp G. (2012). Type II secretion in *Yersinia*—A secretion system for pathogenicity and environmental fitness. Front. Cell. Inf. Microbiol..

[45] Korotkov K.V., Sandkvist M., Hol W.G. (2012). The type II secretion system: Biogenesis, molecular architecture, and mechanism. Nat. Rev. Microbiol..

[46] Nivaskumar M., Francetic O. (2014). Type II secretion system: A magic beanstalk or a protein escalator. Biochim. Biophys. Acta Mol. Cell Res..

[47] Hueck C.J. (1998). Type III protein secretion systems in bacterial pathogens of animals and plants. Microbiol. Mol. Biol. Rev..

[48] Komoriya K., Shibano N., Higano T., Azuma N., Yamaguchi S., Aizawa S.-I. (1999). Flagellar proteins and type III-exported virulence factors are the predominant proteins secreted into the culture media of *Salmonella typhimurium*. Mol. Microbiol..

[49] Cascales E. (2008). The type VI secretion toolkit. EMBO Rep..

[50] Wang J., Brodmann M., Basler M. (2019). Assembly and subcellular localization of bacterial type VI secretion systems. Annu. Rev. Microbiol..

[51] Smith W.P.J., Vettiger A., Winter J., Ryser T., Comstock L.E., Basler M., Foster K.R. (2020). The evolution of the type VI secretion system as a disintegration weapon. PLoS Biol..

[52] Lin J.S., Wu H.H., Hsu P.H., Ma L.S., Pang Y.Y., Ming-Daw Tsai M.D., Lai E.M. (2014). Fha interaction with phosphothreonine of TssL activates Type VI secretion in *Agrobacterium tumefaciens*. PLoS Pathog..

[53] Hernandez R.E., Gallegos-Monterrosa R., Coulthurst S.J. (2020). Type VI secretion system effector proteins: Effective weapons for bacterial competitiveness. Cell. Microbiol..

[54] Leiman P.G., Basler M., Ramagopal O.A., Bonanno J.B., Sauder J.M., Pukatzki S., Burley S.K., Almo S.C., Mekalanos J.J. (2009). Type VI secretion apparatus and phage tail-associated protein complexes share a common evolutionary origin. Proc. Natl. Acad. Sci. USA.

[55] Pukatzki S., Ma A.T., Sturtevant D., Krastins B., Sarracino D., Nelson W.C., Heidelberg J.F., Mekalanos J.J. (2006). Identification of a conserved bacterial protein secretion system in *Vibrio cholerae* using the *Dictyostelium* host model system. Proc. Natl. Acad. Sci. USA.

[56] Bingle L.E.H., Bailey C.M., Pallen M.J. (2008). Type VI secretion: A beginner’s guide. Curr. Opin. Microbiol..

[57] Boyer F., Fichant G., Berthod J., Vandenbrouck Y., Attree I. (2009). Dissecting the bacterial type VI secretion system by a genome wide in silico analysis: What can be learned from available microbial genomic resources?. BMC Genom..

[58] Aschtgen M.S., Bernard C.S., De Bentzmann S., Lloubès R., Cascales E. (2008). SciN is an outer membrane lipoprotein required for type VI secretion in enteroaggregative *Escherichia coli*. J. Bacteriol..

[59] Das S., Chakrabortty A., Banerjee R., Chaudhuri K. (2002). Involvement of in vivo induced icmF gene of *Vibrio cholerae* in motility, adherence to epithelial cells, and conjugation frequency. Biochem. Biophys. Res. Commun..

[60] Weber B., Hasic M., Chen C., Wai S.N., Milton D.L. (2009). Type VI secretion modulates quorum sensing and stress response in *Vibrio anguillarum*. Environ. Microbial..

[61] Filloux A. (2009). The type VI secretion system: A tubular story. EMBO J..

[62] Leng X., Zhu W., Jin J., Mao X. (2011). Evidence that a chaperone-usher-like pathway of *Myxococcus xanthus* functions in spore coat formation. Microbiology.

[63] Hospenthal M.K., Costa T.R.D., Waksman G. (2017). A comprehensive guide to pilus biogenesis in Gram-negative bacteria. Nat. Rev. Microbiol..

[64] Egan A.J.F. (2018). Bacterial outer membrane constriction. Mol. Microbiol..

[65] Szczepaniak J., Press C., Kleanthous C. (2020). The multifarious roles of Tol-Pal in Gram-negative bacteria. FEMS Microbiol. Rev..

[66] Gumbart J., Wiener M.C., Tajkhorshid E. (2007). Mechanics of force propagation in TonB-Dependent outer membrane transport. Biophys. J..

[67] Oke M., Sarra R., Ghirlando R., Farnaud S., Gorringe A.R., Evans R.W., Buchanan S.K. (2004). The plug domain of a neisserial TonB-dependent transporter retains structural integrity in the absence of its transmembrane beta-barrel. FEBS Lett..

[68] Koebnik R. (2005). TonB-dependent trans-envelope signalling: The exception or the rule?. Trends Microbiol..

[69] Flemming H.-C., Wingender J. (2010). The biofilm matrix. Nat. Rev. Microbiol..

[70] Claessen D., Rozen D.E., Kuipers O.P., Søgaard-Andersen L., van Wezel G.P. (2014). Bacterial solutions to multicellularity: A tale of biofilms, filaments, and fruiting bodies. Nat. Rev. Microbiol..

[71] Flemming H.-C., Wingender J., Szewzyk U., Steinberg P., Rice S.A., Kjelleberg S. (2016). Biofilms: An emergent form of bacterial life. Nat. Rev. Microbiol..

[72] Di Martino P. (2018). Extracellular polymeric substances, a key element in understanding biofilm phenotype. AIMS Microbiol..

[73] Cuthbertson L., Mainprize I.L., Naismith J.H., Whitfield C. (2009). Pivotal Roles of the Outer Membrane Polysaccharide Export and Polysaccharide Copolymerase Protein Families in Export of Extracellular Polysaccharides in Gram-Negative Bacteria. Microbiol. Mol. Biol. Rev..

[74] Sperandeo P., Martorana A.M., Polissi A. (2017). Lipopolysaccharide biogenesis and transport at the outer membrane of Gram-negative bacteria. Biochim. Biophys. Acta Mol. Cell Biol. Lipids..

[75] Kumar S.A., Mody K., Jha B. (2007). Bacterial exopolysaccharides—A perception. J. Basic Microbiol..

[76] Whitney J.C., Howell P.L. (2013). Synthase-dependent exopolysaccharide secretion in Gram-negative bacteria. Trends Microbiol..

[77] Low K.E., Howell P.L. (2018). Gram-negative synthase-dependent exopolysaccharide biosynthetic machines. Curr. Opin. Struct. Biol..

[78] Ferreira A.S., Leitão J.H., Silva I.N., Pinheiro P.F., Sousa S.A., Ramos C.G., Moreira L.M. (2010). Distribution of cepacian biosynthesis genes among environmental and clinical *Burkholderia* strains and role of cepacian exopolysaccharide in resistance to stress conditions. Appl. Environ. Microbiol..

[79] Cescutti P., Foschiatti M., Furlanis L., Lagatolla C., Rizzo R. (2010). Isolation and characterisation of the biological repeating unit of cepacian, the exopolysaccharide produced by bacteria of the *Burkholderia cepacia* complex. Carbohydr. Res..

[80] An W., Guo F., Song Y., Gao N., Bai S., Dai J., Wei H., Zhang L., Yu D., Xia M. (2016). Comparative genomics analyses on EPS biosynthesis genes required for floc formation of *Zoogloea resiniphila* and other activated sludge bacteria. Water Res..

[81] Okano C., Koki C., Eri N., Kenichi I., Norihiro K. (2017). Complex flocculation of biofilm-forming bacteria in the presence of flocculating bacteria isolated from activated sludge, transactions of the materials. Res. Soc. Jpn..

[82] Hummel H., Sepers A.B.J., De Wolf L., Melissen F.W. (1988). Bacterial growth on the marine sponge *Halichondria panicea* induced by reduced waterflow rate. Mar. Ecol. Prog. Ser..

[83] Gao N., Xia M., Dai J., Yu D., An W., Li S., Qiu D. (2018). Both widespread PEP-CTERM proteins and exopolysaccharides are required for floc formation of *Zoogloea resiniphila* and other activated sludge bacteria. Environ. Microbiol..

[84] Jarrell K.F., Bayley D.P., Kostyukova A.S. (1996). The archaeal flagellum: A unique motility structure. J. Bacteriol..

[85] Jian H., Wang H., Zeng X., Xiong L., Wang F., Xiao X. (2016). Characterization of the relationship between polar and lateral flagellar structural genes in the deep-sea bacterium *Shewanella piezotolerans WP3*. Sci. Rep..

[86] Ottemann K.M., Lowenthal A.C. (2002). *Helicobacter pylori* uses motility for initial colonization and to attain robust infection. Infect. Immun..

[87] Wadhams G.H., Armitage J.P. (2004). Making sense of it all: Bacterial chemotaxis. Nat. Rev. Mol. Cell. Biol..

[88] Hickman J.W., Tifrea D.F., Harwood C.S. (2005). A chemosensory system that regulates biofilm formation through modulation of cyclic diguanylate levels. Proc. Nat. Acad. Sci. USA.

[89] Strom M.S., Lory S. (1993). Structure-function and biogenesis of the Type Iv Pili. Ann. Rev. Microbiol..

[90] Goosens V.J., Busch A., Georgiadou M., Castagnini M., Forest K.T., Waksman G., Pelicic V. (2017). Reconstitution of a minimal machinery capable of assembling periplasmic type IV pili. Proc. Natl. Acad. Sci. USA.

[91] Choi S.Y., Kim S., Lyuck S., Kim S.B., Mitchell R.J. (2015). High-level production of violacein by the newly isolated *Duganella violaceinigra* str. NI28 and its impact on *Staphylococcus aureus*. Sci. Rep..

[92] Durán N., Justo G.Z., Durán M., Brocchi M., Cordi L., Tasic L., Castro G.R., Nakazato G. (2016). Advances in *Chromobacterium violaceum* and properties of violacein-its main secondary metabolite: A review. Biotechnol. Adv..

[93] Pantanella F., Berlutti C., Passariello S., Sarli S., Morea C., Schippa S. (2007). Violacein and biofilm production. Janthinobacterium Lividum. Appl. Environ. Microbiol..

[94] Venegas F.A., Köllisch G., Diederich M.K., Kaufmann W.E., Bauer A., Bauer S., Chavarría M., Araya J.J., García-Piñeres A.J. (2019). The bacterial product violacein exerts an immunostimulatory effect Via TLR8. Sci. Rep..

[95] Batista A., Moreira A., de Carvalho R., Sales G., Nogueira P., Grangeiro T., Medeiros S., Silveira E., Nogueira N. (2017). Antimicrobial effects of violacein against planktonic cells and biofilms of *Staphylococcus aureus*. Molecules.

[96] Harrington C., Reen F., Mooij M., Stewart F., Chabot J.-B., Guerra A., Glöckner F.O., Nielsen K.F., Gram L., Dobson A.D. (2014). Characterisation of non-autoinducing tropodithietic acid (TDA) production from marine sponge *Pseudovibrio* species. Mar. Drugs.

[97] Campillo-Brocal J.C., Chacón-Verdú M.D., Lucas-Elío P., Sánchez-Amat A. (2015). Distribution in microbial genomes of genes similar to lodA and goxA which encode a novel family of quinoproteins with amino acid oxidase activity. BMC Genom..

[98] Lucas-Elio P., Gomez D., Solano F., Sanchez-Amat A. (2006). The antimicrobial activity of marinocine, synthesized by *Marinomonas mediterranea*, is due to hydrogen peroxide generated by its lysine oxidase activity. J. Bacteriol..

[99] Mai-Prochnow A., Lucas-Elío P., Egan S., Thomas T., Webb J.S., Sanchez-Amat A., Kjelleberg S. (2008). Hydrogen peroxide linked to lysine oxidase activity facilitates biofilm differentiation and dispersal in several Gram-negative bacteria. J. Bacteriol..

[100] Hornung C., Poehlein A., Haack F.S., Schmidt M., Dierking K., Pohlen A., Schulenburg H., Blokesch M., Plener L., Jung K. (2013). The *Janthinobacterium* sp. HH01 genome encodes a homologue of the *V. cholerae* CqsA and *L*. pneumophila LqsA autoinducer synthases. PLoS ONE.

[101] Spirig T., Tiaden A., Kiefer P., Buchrieser C., Vorholt J.A., Hilbi H. (2008). The *Legionella* autoinducer synthase LqsA produces an alpha-hydroxyketone signaling molecule. J. Biol. Chem..

[102] Tiaden A., Spirig T., Hilbi H. (2010). Bacterial gene regulation by α-hydroxyketone signaling. Trends Microbiol..

[103] Pandey D.P. (2005). Toxin-antitoxin loci are highly abundant in free-living but lost from host-associated prokaryotes. Nucleic. Acids Res..

[104] Poulsen B.E., Deber C.M. (2012). Drug efflux by a small multidrug resistance protein is inhibited by a transmembrane peptide. Antimicrob. Agents Chemother..

