# Supplementary material for: Genome Analysis of the Janthinobacterium sp. Strain SLB01 from the Diseased Sponge of the Lubomirskia baicalensis
Source: Curr Issues Mol Biol. 2021 Dec 11;43(3):2220–37. doi: 10.3390/cimb43030156 (PMC8929069; doi:10.3390/cimb43030156)
Supplement: Supplementary file 1 [file cimb-43-00156-s001.zip › cimb-1449216-supplementary/Figure S1. ANI percentage identity of closer species 5 Dec.pdf]

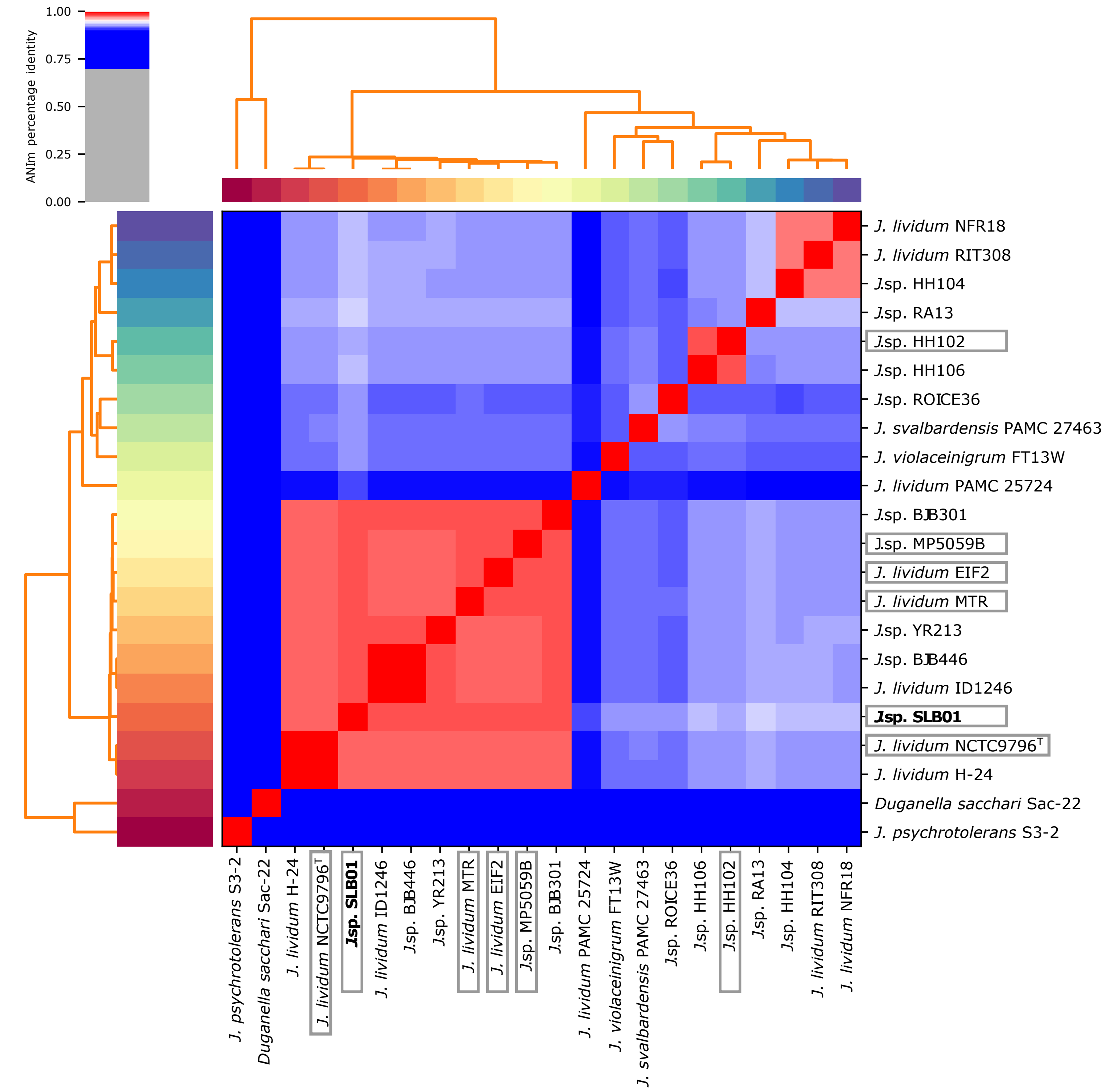

Figure S1. Average Nucleotide % Identity (ANI) comparisons between Janthinobacterium sp. SLB01 and close species with Duganella sacchari Sac-22 as outgroup
