# Supplementary material for: Genome Analysis of the Janthinobacterium sp. Strain SLB01 from the Diseased Sponge of the Lubomirskia baicalensis
Source: Curr Issues Mol Biol. 2021 Dec 11;43(3):2220–37. doi: 10.3390/cimb43030156 (PMC8929069; doi:10.3390/cimb43030156)
Supplement: Supplementary file 1 [file cimb-43-00156-s001.zip › cimb-1449216-supplementary/Table S9. Conservative and variable hydrolytic proteins of Janthinobacterium sp. SLB01.pdf]

Table S9. Conservative and variable hydrolytic proteins in *Janthinobacterium* sp. SLB01

We compared the set of hydrolytic proteins for *Janthinobacterium* sp. SLB01 and closer species in Table S8. Here in Table S9 we divided these proteins to conservative (common for group of closer species) and variable, specific for *Janthinobacterium* sp. SLB01, which have no homologs in genomes of closer species.

***Janthinobacterium* sp. SLB01**		Signal peptide type *
Locus Tag	Annotation	
	Conservative proteins	
F3B38_RS11740	alpha-amylase	SP
F3B38_RS02140	caspase family	SP
F3B38_RS10600	chitinase	–
F3B38_RS15505	chitinase	–
F3B38_RS21655	chitinase	LIPO
F3B38_RS10605	chitinase C-terminal domain-containing	LIPO
F3B38_RS12205	hemolysin III family	–
F3B38_RS00660	ShlB/FhaC/HecB family hemolysin secretion/activation	SP
F3B38_RS01330	ShlB/FhaC/HecB family hemolysin secretion/activation	SP
F3B38_RS22200	ShlB/FhaC/HecB family hemolysin secretion/activation	SP
F3B38_RS08250	patatin-like phospholipase family	–
F3B38_RS09905	patatin-like phospholipase family	–
F3B38_RS11790	patatin-like phospholipase family	–
F3B38_RS02525	patatin-like phospholipase family	SP
F3B38_RS05610	patatin-like phospholipase family	LIPO
F3B38_RS12320	patatin-like phospholipase family	LIPO
F3B38_RS06110	phospholipase	SP
F3B38_RS13770	phospholipase	–
F3B38_RS16045	phospholipase A	–
F3B38_RS07965	phospholipase D family	–
F3B38_RS10925	phospholipase D family	LIPO
F3B38_RS14055	phospholipase D family	SP
F3B38_RS18195	triacylglycerol lipase	SP
F3B38_RS11605	U32 family peptidase	–
F3B38_RS11600	U32 family peptidase collagenase	–
	Variable proteins	
F3B38_RS18770	phospholipase C, phosphocholine-specific	TAT
F3B38_RS22070	alpha/beta hydrolase	–
F3B38_RS15410	alpha/beta hydrolase	SP
F3B38_RS15580	caspase family protein	–
F3B38_RS15585	caspase family protein	–
F3B38_RS17830	caspase family protein	–
F3B38_RS06825	family 43 glycosylhydrolase	SP
F3B38_RS02090	glycoside hydrolase family 127 protein	SP
F3B38_RS24650	glycoside hydrolase family 3 C-terminal domain-containing protein	–
F3B38_RS13215	glycoside hydrolase family 32 protein	–
F3B38_RS04520	S8 family serine peptidase	–
F3B38_RS11800	serine hydrolase	SP
F3B38_RS08740	SGNH/GDSL hydrolase family protein	–
F3B38_RS17735	SOS response-associated peptidase family	–
F3B38_RS17775	SOS response-associated peptidase family	–
F3B38_RS13500	trypsin-like peptidase domain-containing	SP

* SP "standard" secretory signal peptides transported by the Sec translocon and cleaved by Signal Peptidase I (Lep); LIPO: lipoprotein signal peptides transported by the Sec translocon and cleaved by Signal Peptidase II (Lsp); TAT: Tat signal peptides transported by the Tat translocon and cleaved by Signal Peptidase I (Lep); The dash “–” means no signal peptide was detected.
